# A Comprehensive Analysis of the ceRNA Network and Hub Genes in Avian Leukosis Virus Subgroup J and Infectious Bursal Disease Virus Superinfection

**DOI:** 10.3390/ani14233449

**Published:** 2024-11-28

**Authors:** Sheng Chen, Huijuan Xu, Tingxi Pan, Yu Nie, Xinheng Zhang, Feng Chen, Qingmei Xie, Weiguo Chen

**Affiliations:** 1 State Key Laboratory of Swine and Poultry Breeding Industry & Heyuan Branch, Guangdong Provincial Laboratory of Lingnan Modern Agricultural Science and Technology, College of Animal Science, South China Agricultural University, Guangzhou 510642, China; chens@scau.edu.cn (S.C.); sunnyxu20181@outlook.com (H.X.); txpan@scau.stu.edu.cn (T.P.); thcscau@163.com (Y.N.); xhzhang@scau.edu.cn (X.Z.); fengch@scau.edu.cn (F.C.); qmx@scau.edu.cn (Q.X.); 2 Guangdong Provincial Key Lab of AgroAnimal Genomics and Molecular Breeding, College of Animal Science, South China Agricultural University, Guangzhou 510642, China; 3 Guangdong Engineering Research Center for Vector Vaccine of Animal Virus, Guangzhou 510642, China

**Keywords:** ALV-J, IBDV, superinfection, circRNA/lncRNA-miRNA-mRNA network

## Abstract

In the realm of poultry production, viral superinfections pose significant challenges, causing substantial economic losses worldwide. Among these, avian leukosis virus subgroup J (ALV-J) and infectious bursal disease virus (IBDV) are particularly concerning as they frequently lead to superinfections in chicken, further exacerbating production losses and health complications. Our previous research delved into the pathogenicity and immunosuppressive effects of these superinfections through in vitro and in vivo analyses. Yet, the underlying key genes and pathways governing this phenomenon remained elusive. In this study, we randomly selected three chickens at 21 days post infection from each treatment group (ALV-J, IBDV, ALV-J+IBDV, and control group) to collect the bursa of Fabricius samples for full transcriptome analysis. Utilizing these data, we constructed a comprehensive circRNA/lncRNA-miRNA-mRNA network which elucidated both synergistic and specific activations during the superinfection. Notably, three pivotal genes (FILIP1L, DCX, and MYPN) were pinpointed in datasets reflecting synergistic activations. Conversely, four other genes (STAP, HKR6, XKR4, and TLR5) emerged in datasets associated with specific activations. Further exploration revealed diverse significant GO terms and pathways associated with both synergistic and distinct activation processes. These ceRNA network and core genes potentially wield substantial influence over the synergistic or specific activation of tumorigenesis and pathogenesis induced by ALV-J and IBDV. These findings could help develop targeted therapies and improve disease control in poultry, reducing economic losses.

## Simple Summary

Viral superinfections in poultry can seriously impact chicken health and cause major financial losses. Two viruses, avian leukosis virus subgroup J (ALV-J) and infectious bursal disease virus (IBDV), are especially concerning because they often infect chickens together, leading to worsened health issues and reduced productivity. Previous research has studied these effects, but the specific genes and pathways driving this problem were unknown. In this study, we analyzed immune organ samples from chickens infected with either ALV-J, IBDV, or both. Using these data, we mapped out complex networks among the RNA molecules to reveal how these viruses interact both by working together and through individual responses. We identified the key genes and pathways involved in these processes. This insight could support new methods to control these infections, enhancing poultry health and lowering economic losses.

## 1. Introduction

Superinfection, characterized by at least two distinct pathogens simultaneously infecting a host, holds significant implications for various aspects, such as virulence evolution, genetic diversity, epidemiological understanding, and the development of control strategies [1,2]. Superinfections have become widespread in various animal groups in recent decades [3,4]. This multifaceted nature renders the traditional reductionist approach to host–pathogen interactions ill-suited for studying singular infections [5]. At present, the majority of superinfection studies remain at the phenotypic research level [6]. The task of pinpointing crucial differentially expressed genes during superinfection events is complicated by the synergistic effects of multiple factors. We have developed a novel strategy which characterizes the pathogenic effects of superinfection by studying both synergistic and specific activations during the superinfection process. Synergistic activation refers to genes or pathways which can be activated by either virus individually, with superinfection significantly enhancing their expression levels. In contrast, specific activation pertains to genes or pathways which are uniquely activated only in the presence of both viruses simultaneously. Both models reveal the infection of pathogenic characteristics from different angles.

In the poultry industry, avian leukosis virus subgroup J (ALV-J) can induce tumorigenesis and immunosuppression in infected chickens, leading to substantial financial setbacks [7]. Similar to ALV-J, infectious bursal disease virus (IBDV) is also known to trigger tumor development in young chicks [8]. Notably, ALV-J and IBDV often cause superinfections in poultry production [9]. In a previous study, we conducted a comprehensive analysis of the pathogenicity and immunosuppressive activity of superinfections with ALV-J and IBDV in vitro and in vivo [10]. The findings indicated that superinfection synergistically enhances tumorigenesis and disease progression. However, the key genes or pathways regulating this process remain unclear.

Competing endogenous RNAs (ceRNAs) are a class of non-coding RNAs which interact with microRNAs (miRNAs) to regulate gene expression. According to the ceRNA hypothesis, various RNA species can compete for binding to shared miRNAs, thereby influencing the levels of target mRNAs [11,12]. This interaction can modulate a range of biological processes, including cell differentiation, proliferation, and apoptosis. The construction of a ceRNA network is based on three principal criteria: (1) a targeting relationship between miRNAs and potential ceRNAs, accompanied by a negative correlation in their expression patterns; (2) a positive correlation in the expression levels among candidate ceRNAs; and (3) the degree of enrichment of candidate ceRNAs binding to a specific miRNA. In the context of viral infections, ceRNAs may play a critical role in regulating the host’s response to pathogens, potentially affecting the viral life cycle and the pathogenesis of related diseases [13]. Given their regulatory capabilities, ceRNAs may significantly influence the synergistic and specific activation of tumorigenesis and pathogenesis induced by ALV-J and IBDV.

This study aims to investigate the regulatory networks involved in ALV-J and IBDV superinfection by analyzing full transcriptome data to identify the key hub genes and associated pathways. To achieve this, a circRNA/lncRNA-miRNA-mRNA network is constructed through comprehensive analysis of the synergistic and specific activations during the superinfection process. Three hub genes (FILIP1L, DCX, and MYPN) are identified in synergistic activation datasets, while four hub genes (STAP, HKR6, XKR4, and TLR5) are identified in specific activation datasets. Additionally, different significantly enriched GO terms and pathways are identified during the processes of synergistic and characteristic activation. We further validate the differential expression of key hub genes and their targeted relationships with miRNAs. These hub genes may have a significant influence on the synergistic or specific activation of tumorigenesis and pathogenesis by ALV-J and IBDV.

## 2. Materials and Methods

### 2.1. Ethics Statement

Institutional and national guidelines for the use and care of laboratory animals were closely followed. All animal experiments were performed following the guidelines of the South China Agricultural University Animal Care and Use Committee (permit no. SCAU2021b020). This study was performed in positive pressure, high-efficiency, particulate air-filtered stainless steel isolators with an enclosed and ventilated environment, and feed and water were provided ad libitum.

### 2.2. Virus and Animals

The ALV-J strain SCAU-HN06 was a generous gift from Professor Liao of South China Agricultural University. IBDV strain 801 (cell-adapted strain) was stored in our laboratory. Virus titers were calculated using the Reed–Muench formula to calculate the 50% tissue cultural infective dose (TCID_50_) per milliliter. One-day-old SPF chicks (White Leghorn), including both hen chicks and cock chicks, were purchased from Xinxing Dahuanong Poultry Eggs Co., Ltd. (Yunfu, China).

### 2.3. Animal Experimental Design and Sample Collection

**Figure 1:**
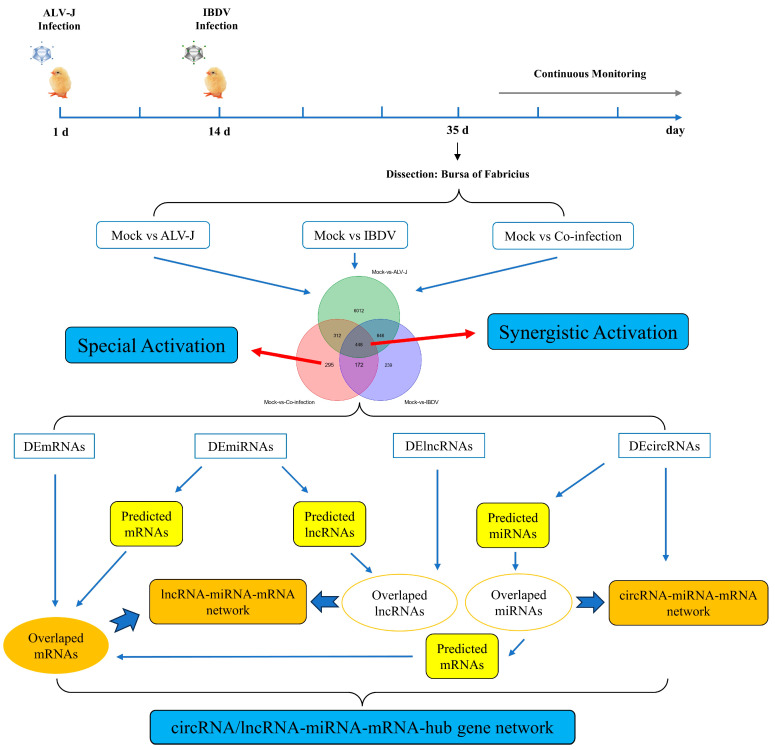
Workflow of bioinformatics analysis.

A total of 220 one-day-old SPF chicks were randomly assigned to four groups: ALV-J, IBDV, ALV-J+IBDV, and a control group. At 1 day of age, the chicks in the ALV-J and ALV-J+IBDV groups were inoculated via intra-abdominal injection with 100 µL of ALV-J at a dose of 10^3.7^ TCID50. At 14 days of age, the chicks in the IBDV and ALV-J+IBDV groups were similarly inoculated with 100 µL of IBDV at a dose of 10^3.6^ ELD50. The control group was given 100 µL of PBS at both day 1 and day 14, following the protocol described in a previous study [10]. At 21 days post infection (35 days of age), three chickens from each group were randomly selected for the collection of bursa of Fabricius, which were then sent to Guangzhou Genedenovo Biotechnology Co., Ltd. (Guangzhou, China) for full transcriptome high-throughput sequencing. The specific groups and treatments are detailed in Figure 1.

### 2.4. Differentially Expressed mRNA, miRNA, circRNA, and lncRNA

To detect differentially expressed transcripts among the samples, the edgeR package (http://www.bioconductor.org/packages/release/bioc/html/edgeR.html, accessed on 18 November 2024) was utilized, where mRNAs, lncRNAs, and circRNAs with a fold change ≥2 and an FDR <0.05 were deemed significant DEGs, while miRNAs required a fold change ≥2 and *p* < 0.05 for significance.

### 2.5. miRNA Target Prediction

For the samples, a triad of computational tools—mireap, miRanda, and TargetScan—was harnessed to discern the miRNA targets. Information pertaining to miRNA sequences and their respective families was gleaned from the TargetScan online repository (accessible at http://www.targetscan.org/, accessed on 18 November 2024).

### 2.6. Construction of ceRNA Network

In the context of ceRNAs being communally regulated by miRNAs, ascertaining the target genes for differentially expressed miRNAs represents an integral preliminary step in exploring ceRNA regulatory networks. The Spearman Rank correlation coefficient (SCC) was employed to assess the expression correlation between mRNA-miRNA, lncRNA-miRNA, or circRNA-miRNA pairs. Pairs exhibiting an SCC of less than −0.7 were deemed to be negatively co-expressed lncRNA–miRNA pairs, mRNA-miRNA pairs, or circRNA-miRNA pairs, with mRNA, lncRNA, and circRNA identified as miRNA target genes and all RNAs demonstrating differential expression.

The Pearson correlation coefficient (PCC) was utilized to evaluate the expression correlation between lncRNA-mRNA or circRNA-mRNA pairs. Pairs characterized by a PCC exceeding 0.9 were recognized as co-expressed lncRNA-mRNA pairs or circRNA-mRNA pairs. In these pairs, both mRNA and lncRNA or both mRNA and circRNA were targeted and exhibited negative co-expression with a shared miRNA.

### 2.7. Visualization of ceRNA Network

The assembly of the lncRNA-miRNA-mRNA network was accomplished by amalgamating all previously identified co-expression competing triplets, which were subsequently visualized using Cytoscape software version 3.6.0, available at http://www.cytoscape.org/, accessed on 18 November 2024.

### 2.8. ceRNA Connectivity Analysis

Within the ceRNA network, RNA connectivity was defined by the count of co-expressed targeted miRNAs. Consequently, ceRNAs exhibiting the highest connectivity were considered hub genes, which are deemed more crucial within biological networks.

### 2.9. Functional Enrichment Analysis

To evaluate functional enrichment, Gene Ontology (GO) biological processes term and Kyoto Encyclopedia of Genes and Genomes (KEGG) pathway analyses of mRNAs within the ceRNA network were conducted using Cytoscape. GO enrichment analysis reveals all GO terms which are significantly enriched in ceRNAs compared with the genome background and filters the ceRNAs associated with specific biological functions. Initially, all ceRNAs were mapped to GO terms in the Gene Ontology database (accessible at http://www.geneontology.org/, accessed on 18 November 2024). The gene counts were tallied for each term, and GO terms significantly enriched in ceRNAs relative to the genomic background were determined using the hypergeometric test.

Genes typically interact to execute certain biological functions. Pathway-based analysis aids in the deeper understanding of gene functions. KEGG is a principal public database related to pathways (available at http://www.kegg.jp/kegg/, accessed on 18 November 2024). Pathway enrichment analysis pinpointed the metabolic pathways or signal transduction pathways which were significantly enriched in ceRNAs when contrasted with the entire genome background.

### 2.10. Reverse Transcription and Quantitative PCR (qRT-PCR)

The total RNA was reverse transcribed into cDNA using a Prime-Script™ cDNA synthesis kit (Takara Bio, Inc., Shanghai, China), while miRNAs were extracted using an miRNA Isolation Kit (Life Technologies, Carlsbad, CA, USA) according to the manufacturer’s protocol. The relative expression of lncRNAs, miRNAs, and mRNAs was evaluated using qRT-PCR. The primers mentioned above for qRT-PCR are listed in Supplementary Table S1. Then, qRT-PCR was performed on a CFX96 system (Bio-Rad Laboratories, Inc., Berkeley, CA, USA) using Power SYBR Green PCR Master Mix (Roche Diagnostic, Indianapolis, IN, USA) according to the manufacturer’s protocol. Relative gene expression was calculated using the 2^−ΔΔCT^ method.

### 2.11. Statistical Analysis

GraphPad Prism version 8.1 (GraphPad Software, Inc., San Diego, CA, USA) was used for statistical analysis. The values reported in each graph are expressed as the mean ± standard error of the mean (S.E.M.) of at least three independent experiments. One-way ANOVA was used to test the differences between different groups. We considered *p* < 0.05 to be statistically significant. (* *p* < 0.05; ** *p* < 0.01; *** *p* < 0.001).

## 3. Results

### 3.1. Identification of Differentially Expressed circRNAs (DEcircRNAs), lncRNAs (DElncRNAs), miRNAs (DEmiRNAs), and mRNAs (DEmRNAs) in Superinfection of ALV-J and IBDV

Our previous research results indicated that superinfection with ALV-J and IBDV synergistically causes pathogenicity by mutually enhancing viral replication, leading to more severe immunosuppression than infections with ALV-J or IBDV alone [10]. However, the molecular mechanisms underlying this outcome are still unclear. Consequently, we randomly selected three chickens at 21 days post infection from each group (ALV-J, IBDV, ALV-J+IBDV, and control group) to collect bursa of Fabricius samples for full transcriptome analysis using high-throughput sequencing.

As illustrated in Figure 1, this strategy was employed to analyze the competing endogenous RNA (ceRNA) regulatory network during the superinfection process with ALV-J and IBDV, focusing on both synergistic and specific activations (Figure 1). In addressing synergistic activation, we filtered differentially expressed mRNAs, miRNAs, lncRNAs, and circRNAs which exhibited synergistic activation for integrative analysis, subsequently constructing a circRNA/lncRNA-miRNA-mRNA hub gene network. For specific activation, differentially expressed RNAs which were characteristically activated were selected, and a similar gene network analysis was conducted.

To ensure data quality, it was essential to perform data filtering on the raw data before information analysis to minimize interference from invalid data. We utilized FastP for quality control on the raw reads generated from sequencing, filtering out low-quality data to obtain clean reads (Supplementary Table S2). Subsequently, we used the short read alignment tool Bowtie2 to align the clean reads with the ribosomal database of the species, removing reads which mapped to ribosomes without allowing for mismatches. The unmapped reads which remained were then used for subsequent transcriptomic analysis (Supplementary Table S2).

For the differential expression analysis of various RNA types based on expression levels, we selected mRNAs, lncRNAs, and circRNAs with an FDR < 0.05 and |log2FC| > 1 and miRNAs with *p* < 0.05 and |log2FC| > 1 as significantly differentially expressed RNAs. In terms of differential mRNAs, compared with the mock group, the ALV-J group had a total of 70 genes downregulated and 378 genes upregulated, and the IBDV group had 87 genes downregulated and 361 genes upregulated, while the superinfection group had 90 genes downregulated and 358 genes upregulated (Figure S1A and Supplementary Table S3). The differential miRNAs are presented in Figure S1B and Supplementary Table S3, the differential lncRNAs are shown in Figure S1C and Supplementary Table S3, and the differential circRNAs are shown in Figure S1D and Supplementary Table S3.

**Figure 2:**
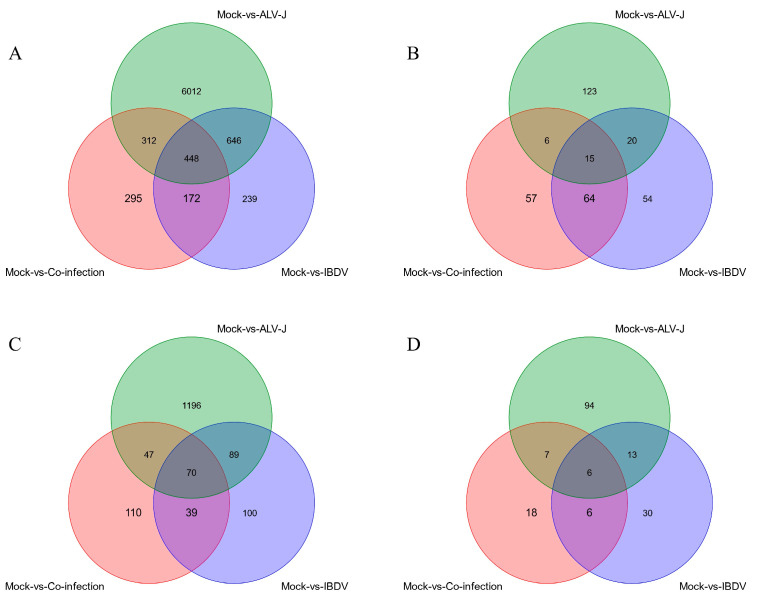
Venn diagram of differentially expressed RNAs. (**A**) mRNAs. (**B**) miRNAs. (**C**) lncRNAs. (**D**) circRNAs.

To identify genes which play a pivotal role during the superinfection process, the strategy delineated above was employed to analyze differentially expressed genes across the comparison groups (Figure 2). Among these, there were 448 mRNAs, 15 miRNAs, 70 lncRNAs, and 6 circRNAs synergistically activated during superinfection (Supplementary Table S4). For signature activation in superinfection, there were 295 mRNAs, 57 miRNAs, 110 lncRNAs, and 18 circRNAs (Supplementary Table S5). Tailored circRNA/lncRNA-miRNA-mRNA interaction networks were constructed for both the synergistic and signature activation processes observed in superinfection, followed by subsequent functional analysis.

### 3.2. Construction of ceRNA Network During Superinfection with Synergistic Activation

**Figure 3:**
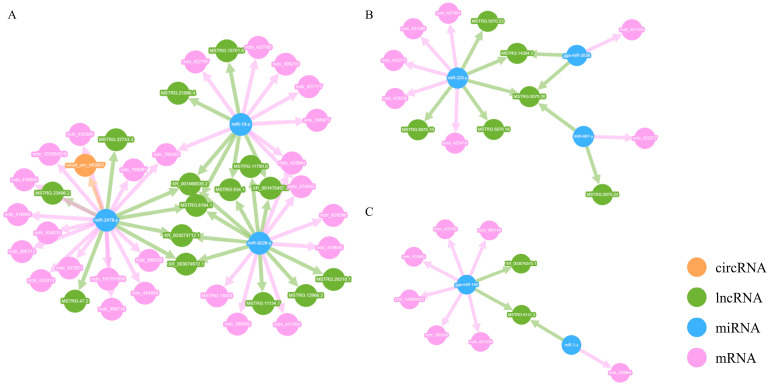
The circRNA/lncRNA-miRNA hub gene network. Construction of ceRNA network during superinfection with synergistic activation. (**A**) Interaction network showing the regulatory relationships among circRNAs, lncRNAs, miRNAs, and mRNAs; (**B**) Key regulatory network highlighting miRNA-mediated lncRNA and mRNA interactions; (**C**) Subnetwork emphasizing the regulation of specific miRNA hubs connecting lncRNAs and mRNAs.

In accordance with the ceRNA hypothesis, circRNAs or lncRNAs can inhibit miRNAs, which themselves repress mRNAs. Thus, after filtering out mismatched RNA interaction pairs, a circRNA/lncRNA-miRNA hub gene network was formed, consisting of 1 circRNA, 23 lncRNAs, 8 miRNAs, and 40 mRNAs (Figure 3).

**Figure 4:**
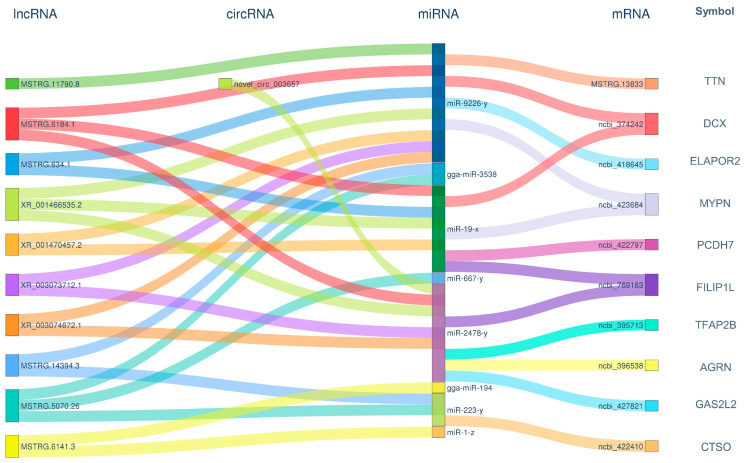
A ceRNA connectivity Sankey diagram during superinfection with synergistic activation. We created a Sankey diagram of the top 10 mRNA/lncRNA/circRNA paths with the highest connectivity, illustrating their targeting regulatory relationships with miRNAs. The diagram visualizes the interactions between circRNAs, lncRNAs, miRNAs, and mRNAs, showing the flow of regulatory relationships. Each node represents a specific RNA molecule, with the width of the connecting lines indicating the strength or frequency of interactions. The left side displays circRNAs and lncRNAs, which act as potential ceRNAs, while miRNAs are placed in the center, mediating the regulatory interactions. The right side shows the target mRNAs influenced by ceRNA interactions. The diagram highlights how different RNA species compete for shared miRNAs, affecting gene expression. The colors correspond to different RNA types, and the thickness of the connections reflects the enrichment level of the interaction.

In regulatory networks, nodes with high connectivity often hold significant biological importance; these genes are referred to as hub genes. In the ceRNA regulatory network, the connectivity of an RNA molecule (lncRNA/mRNA/circRNA) is defined as the number of miRNA molecules with which it has a targeting regulatory relationship. RNA molecules with higher connectivity possess a greater potential regulatory capability. For the top 10 mRNA/lncRNA/circRNA paths in terms of connectivity, a Sankey diagram was created to illustrate their targeting regulatory relationships with miRNAs (Figure 4). Among them, three hub genes of significant biological importance were identified, including FILIP1L (ncbi_769163), DCX (ncbi_374242), and MYPN (ncbi_423684). These hub genes may play an important role in the synergetic activation of tumorigenesis and pathogenesis by ALV-J and IBDV.

### 3.3. Biological Functions of DEmRNAs in the ceRNA Network During Superinfection with Synergistic Activation

**Figure 5:**
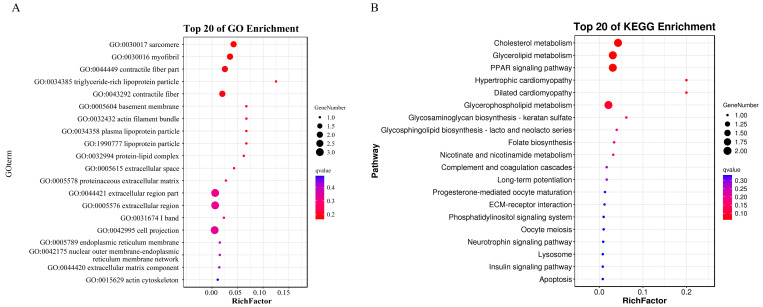
Biological functions of DEmRNAs in the ceRNA network during superinfection with synergistic activation. (**A**) GO terms. (**B**) KEGG pathway. The top 20 GO and KEGG terms with the smallest Q-values are presented. The y axis represents the GO and KEGG terms, and the x axis shows the enrichment factor (the ratio of the number of differentially expressed genes in a GO or KEGG term to the total number of genes in that term). The size of the dots indicates the number of genes, and the color gradient from red to yellow reflects the Q-value, with red indicating smaller Q-values.

GO annotation and KEGG functional enrichment analyses were performed on the mRNAs in the ceRNA regulatory network, revealing significantly enriched gene functions and pathways within the ceRNA regulatory network. This provides clues for selecting the target genes in the next step. The GO analysis results show that the most enriched biological processes (BPs) mainly included cellular process and biological regulation. The cell, cell part, and organelle were the most enriched cellular components (CCs). According to the molecular function (MF), these DEmRNAs were mainly enriched in terms of binding, catalytic activity, and the molecular function regulator (Figure 5A and Supplementary Table S6). In KEGG pathway enrichment analysis, these DEmRNAs are mainly involved in the cholesterol metabolism, glycerolipid metabolism, PPAR signaling pathway, and glycerophospholipid metabolism (Figure 5B and Supplementary Table S7).

### 3.4. Construction of ceRNA Network During Superinfection with Specific Activation

**Figure 6:**
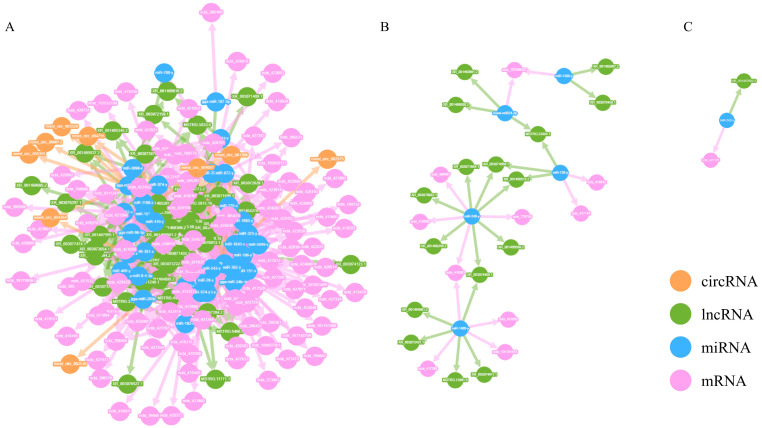
The circRNA/lncRNA-miRNA hub gene network. Construction of ceRNA network during superinfection with specific activation. (**A**) Interaction network showing the regulatory relationships among circRNAs, lncRNAs, miRNAs, and mRNAs; (**B**) Key regulatory network highlighting miRNA-mediated lncRNA and mRNA interactions; (**C**) Subnetwork emphasizing the regulation of specific miRNA hubs connecting lncRNAs and mRNAs.

**Figure 7:**
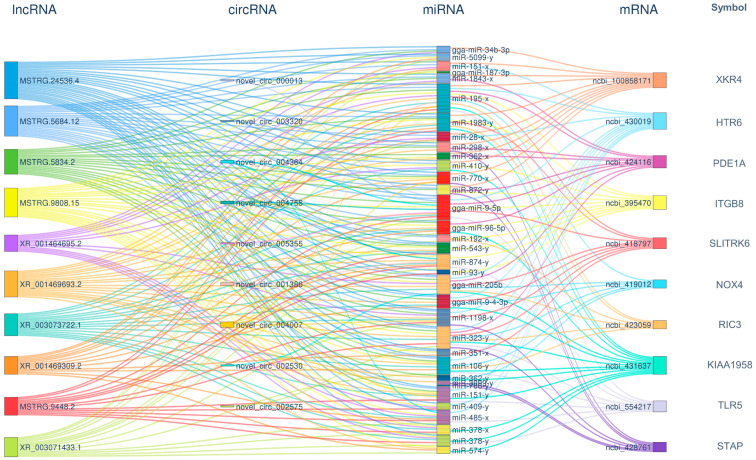
ceRNA connectivity Sankey diagram during superinfection with specific activation. The diagram visualizes the interactions between circRNAs, lncRNAs, miRNAs, and mRNAs, showing the flow of regulatory relationships. Each node represents a specific RNA molecule, with the width of the connecting lines indicating the strength or frequency of interactions. The left side displays circRNAs and lncRNAs, which act as potential ceRNAs, while miRNAs are placed in the center, mediating the regulatory interactions. The right side shows the target mRNAs influenced by ceRNA interactions. The diagram highlights how different RNA species compete for shared miRNAs, affecting gene expression. The colors correspond to different RNA types, and the thickness of the connections reflects the enrichment level of the interaction.

In response to the uniquely activated genes observed during the superinfection process, a ceRNA regulatory network was also established. This network was composed of 9 circRNAs, 63 lncRNAs, 40 miRNAs, and 108 mRNAs (Figure 6). A Sankey diagram was similarly employed to depict their targeting regulatory interactions with miRNAs (Figure 7). Notably, the hub genes exhibiting the highest connectivity includeed STAP (ncbi_428761), HTR6 (ncbi_430019), XKR4 (ncbi_100858171), and TLR5 (ncbi_554217). The hub genes likely had a significant influence on the specific activation of tumorigenesis and pathogenesis by ALV-J and IBDV.

### 3.5. Biological Functions of DEmRNAs in the ceRNA Network During Superinfection with Specific Activation

**Figure 8:**
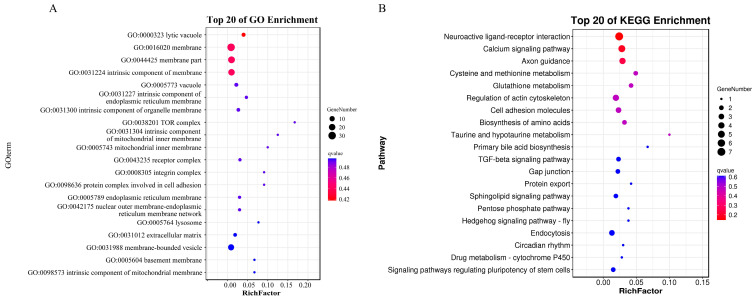
Biological functions of DEmRNAs in the ceRNA network during superinfection with specific activation. (**A**) GO terms. (**B**) KEGG pathway. The top 20 GO and KEGG terms with the smallest Q-values are presented. The y axis represents the GO and KEGG terms, and the x axis shows the enrichment factor (the ratio of the number of differentially expressed genes in a GO or KEGG term to the total number of genes in that term). The size of the dots indicates the number of genes, and the color gradient from red to yellow reflects the Q-value, with red indicating smaller Q-values.

The GO analysis outcomes indicate that the most significantly enriched BPs primarily encompassed cellular processes, single-organism processes, and biological regulation. As for the CCs, cells, cell parts, and organelles were found to be the most enriched categories. Regarding MFs, these DEmRNAs were predominantly associated with binding, catalytic activity, and molecular function regulator activity (Figure 8A and Supplementary Table S8). In the KEGG pathway enrichment analysis, these DEmRNAs were mainly implicated in neuroactive ligand–receptor interaction, the calcium signaling pathway, and axon guidance (Figure 8B and Supplementary Table S9).

### 3.6. Verification of Hub Genes, miRNAs, and circRNAs in the ceRNA Network

**Figure 9:**
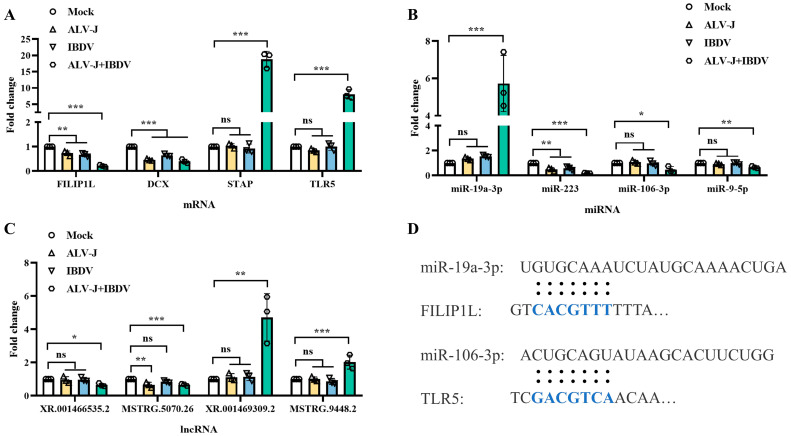
qPCR identification and miRNA-mRNA predication. (**A**) Identification of DEmRNAs during superinfection. (**B**) Identification of DEmiRNAs during superinfection. (**C**) Identification of DElncRNAs during superinfection. (**D**) miRNA-mRNA prediction used by miRNA target prediction database. * *p* < 0.05; ** *p* < 0.01; *** *p* < 0.001; ns *p* > 0.05.

To validate the sequencing results, we employed a rigorous approach which involved the random selection of four differentially expressed transcripts from the ceRNA network for qPCR analysis. As illustrated in Figure 9, the gene expression patterns of the differentially expressed genes, miRNAs, and lncRNAs chosen from each group were not only statistically significant but also exhibited trends which aligned with the sequencing data. This congruence between the qPCR validation and sequencing outcomes underscores the reliability and precision of our findings. In addition, we also used the miRNA target prediction database to predict the targeting relationship between candidate miRNAs and mRNAs.

## 4. Discussion

Our findings provide novel insights into the molecular mechanisms underlying the superinfection of ALV-J and IBDV. Through transcriptome analysis of bursae of Fabricius collected from SPF chickens (21 days post infection), we identified both synergistic and specific activation patterns within the constructed circRNA/lncRNA-miRNA-mRNA network. These networks revealed key regulatory elements which potentially drive the pathogenesis and immune response during superinfection.

During the process of synergistic activation by ALV-J and IBDV superinfection, three key hub genes were identified. Filamin A interacting protein 1-llike (FILIP1L) is a novel tumor suppressor-like protein which has its expression downregulated in various cancers, like colorectal cancer [14], ovarian cancer [15], and lung cancer [16]. FILIP1L-mediated cell apoptosis, epithelial-mesenchymal transition, and extracellular matrix synthesis aggravate multiple tumorigenesis [17]. Importantly, the function of FILIP1L’s decrease in enhancing tumor invasion and metastasis is mainly achieved by promoting the Wnt/β-catenin signaling pathway [18]. It is interesting to note that ALV-J induced Wnt signaling activation, which is reported to have caused tumor generation [19,20]. In this study, we found that separate ALV-J infections or IBDV infections can induce a decrease in FILIP1L expression, two kinds of virus superinfection more significantly lowered FILIP1L expression and prompt FILIP1L cutting, and repeated infection-induced tumor enhancement is closely related to the formation. Doublecortin (DCX) is a key regulatory protein in the JNK signaling pathway and closely associated with cell survival and apoptotic processes [21]. Doublecortin-like kinase 1 is an important target in various virus infection and tumorgenesis processes [22]. Importantly, Doublecortin-like kinase 1 was identified as a cancer stem cell marker in ALV-J-infected cells, which interacts with the surface protein of ALV-J to promote virus replication, activate the epithelial-mesenchymal transition, and accelerate cell proliferation, enabling ALV-J to obtain metastatic ability [23]. These reports further suggest that DCX may play an important role in the synergistic tumorigenesis of ALV-J and IBDV. MYPN, with TTN (MSTRG_13833), another identified significantly different mRNA, may have a common function which has been reported in both human disease and porcine growth and development [24,25].

A total of four key hub genes were identified in the process of specific activation of ALV-J and IBDV superinfection, including STAP, HTR6, XKR4, and TLR5. The signal-transducing adaptor protein (STAP) family, particularly STAP-2, is known to interact with key components of immune signaling pathways, such as the IKK complex, STAT3, STAT5, and MyD88, a critical adaptor in TLR signaling [26,27]. This interaction promotes inflammatory cytokine production and regulates immune responses. In our study, STAP was specifically upregulated during the superinfection of ALV-J and IBDV, suggesting its potential role in amplifying immunosuppressive effects, creating a favorable environment for persistent viral infection and tumorigenic processes. Additionally, our data show that miR-106-3p, a key regulator upstream of TLR5, was significantly downregulated during superinfection. This suggests that TLR signaling may be activated in response to superinfection, as TLR5 plays a pivotal role in pathogen recognition and immune activation [28]. The simultaneous activation of STAP and TLR5 signaling pathways may reflect a complex immune regulatory strategy during superinfection, where initial immune responses are activated but are subsequently modulated to suppress effective immunity, facilitating viral persistence and potential tumorigenesis. These findings provide new insights into how viral infections manipulate immune signaling pathways to enhance pathogenesis and may guide future studies in identifying therapeutic targets for mitigating the effects of such superinfections in poultry.

## 5. Conclusions

In conclusion, our study successfully constructed a comprehensive circRNA/lncRNA-miRNA-mRNA network which revealed the complex interplay of RNA molecules during the superinfection of ALV-J and IBDV. This network elucidated both synergistic and specific activation processes, providing new insights into the molecular mechanisms underlying these viral infections. Notably, we identified three pivotal genes (FILIP1L, DCX, and MYPN) linked to synergistic activation, suggesting their potential roles in enhancing viral pathogenesis. In contrast, four other genes (STAP, HKR6, XKR4, and TLR5) were specifically activated, indicating their involvement in modulating immune responses during superinfection. Our findings highlight significant GO terms and pathways which may drive tumorigenesis and immunosuppression associated with ALV-J and IBDV. These results underscore the importance of further investigation into these key regulatory pathways, which may offer therapeutic targets for mitigating the adverse effects of viral superinfections in poultry.

## Data Availability

All the data generated or analyzed in this study are included in this paper.

## Supplementary Materials

The following supporting information can be downloaded at https://www.mdpi.com/article/10.3390/ani14233449/s1.
