# Supplementary figures and images for: A Comprehensive Analysis of the ceRNA Network and Hub Genes in Avian Leukosis Virus Subgroup J and Infectious Bursal Disease Virus Superinfection
Source: Animals (Basel). 2024 Nov 28;14(23):3449. doi: 10.3390/ani14233449 (PMC11640342; doi:10.3390/ani14233449)

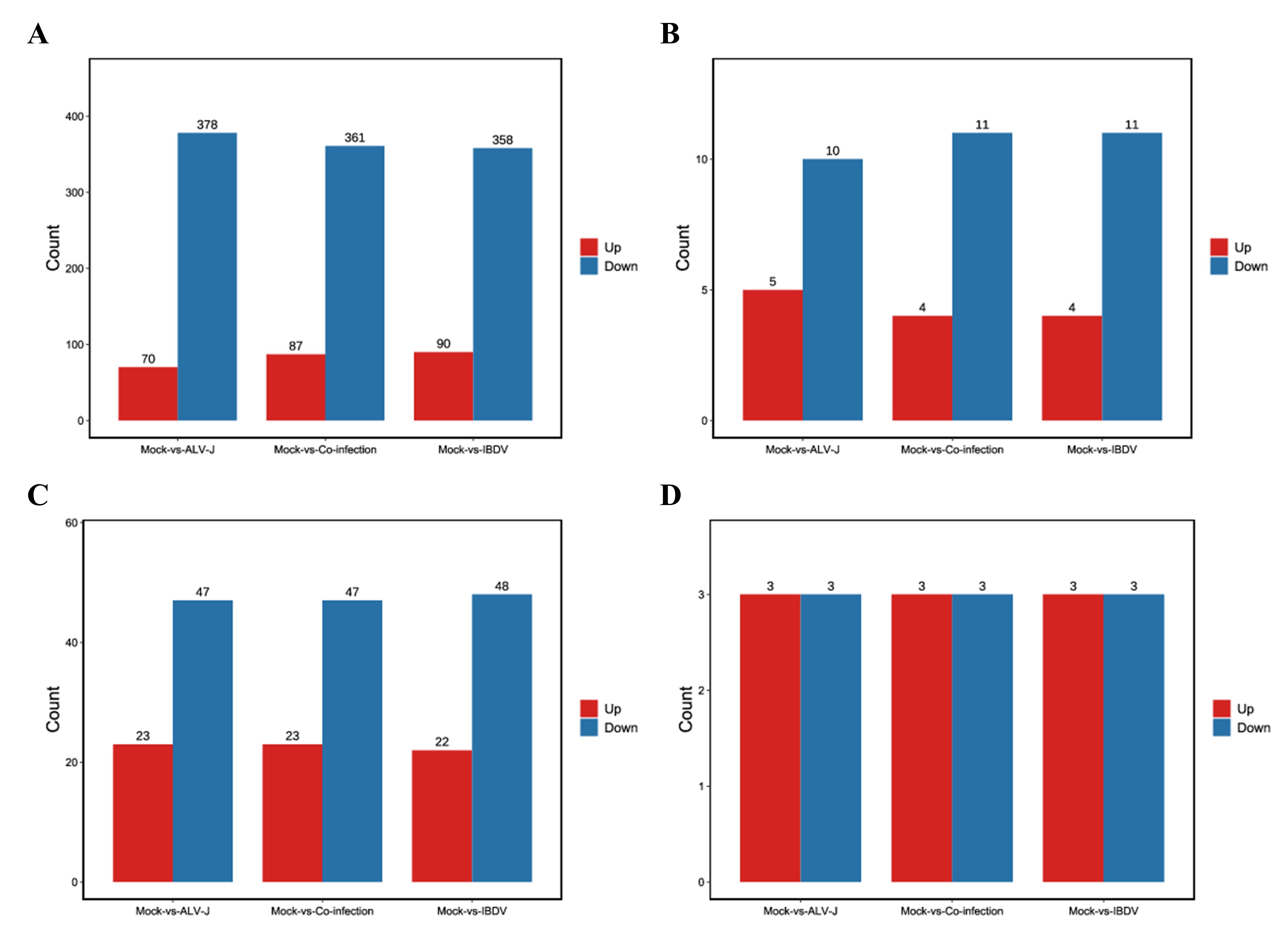

Supplement: Supplementary file 1 [file animals-14-03449-s001.zip › Figure S1.jpg]
